# Toxin protein LukS-PV targeting complement receptor C5aR1 inhibits cell proliferation in hepatocellular carcinoma *via* the HDAC7–Wnt/β-catenin axis

**DOI:** 10.1016/j.jbc.2024.108148

**Published:** 2024-12-28

**Authors:** Lan Shi, Shanshan Zhang, Gan Liu, Zhengchao Nie, Pengsheng Ding, Wenjiao Chang, Yuanyuan Dai, Xiaoling Ma

**Affiliations:** 1 Department of Clinical Laboratory, The First Affiliated Hospital of University of Science and Technology of China (USTC), Division of Life Sciences and Medicine, University of Science and Technology of China, Hefei, China; 2 Department of Medical Oncology, The First Affiliated Hospital of University of Science and Technology of China (USTC), Division of Life Sciences and Medicine, University of Science and Technology of China, Hefei, China

**Keywords:** hepatocellular carcinoma, bacterial toxin, G protein–coupled receptor, cancer therapy, HDAC7, β-catenin, cell proliferation, LukS-PV, C5aR1

## Abstract

Hepatocellular carcinoma (HCC) is one of the common malignant tumors. Complement system has become a new focus of cancer research by changing the biological behavior of cancer cells to influence the growth of cancer. Recent studies reported that the complement C5a–C5aR1 axis can promote the malignant phenotype of multiple tumors through various signaling pathways. LukS-PV (Panton–Valentine), the S component of *Staphylococcus aureus*–secreted PV leucocidin, can also bind C5aR1 specifically. This project aims to investigate the role of LukS-PV on HCC cell proliferation and explore underlying molecular mechanisms. Our findings revealed that LukS-PV targeting C5aR1 inhibited HCC cell proliferation *in vitro* and *in vivo*. Interestingly, we discovered that LukS-PV inhibited the proliferation of HCC cells by upregulating the acetylation level of β-catenin to promote its protein degradation. In addition, histone deacetylase (HDAC)7 identified as a regulator mediates the deacetylation of β-catenin. Furthermore, our results showed that LukS-PV inhibited proliferation in HCC cells by downregulating HDAC7 to promote the degradation of β-catenin through ubiquitin–proteasome system. Collectively, our findings revealed that LukS-PV targeting C5aR1 inhibits HCC cell proliferation through the HDAC7-Wnt/β-catenin axis. These results revealing a novel mechanism that LukS-PV as a bacterial toxin inhibits HCC cell proliferation through epigenetic remodeling by targeting complement receptor C5aR1, suggest the strong potential of LukS-PV as a promising candidate for HCC treatment.

Hepatocellular carcinoma (HCC) is one of the most common malignant tumors of the digestive system. The Global Cancer Statistics 2020 report showed that the annual incidence of new liver cancer ranks it sixth among malignant tumors, whereas the mortality rate ranks it as the third leading cause of cancer deaths worldwide (1). The occurrence and development of liver cancer is a complex process involving multiple factors and complicated gene regulation (2). In recent years, studies have found that molecular targeted therapy has unique advantages in controlling malignant proliferation and metastasis while improving the overall survival rate of HCC, receiving great attention as a new research hotspot (3, 4).

Most HCC develops following long-term chronic inflammation of the liver (5). The complement system participates as an important inflammatory mediator of the immune response (6). In recent years, the complement system has been found to affect tumor growth by changing the biological behavior of cancer cells, becoming a new hotspot in the research of tumor therapy (7, 8). The receptor for complement component C5a, C5aR1, is reportedly highly expressed in a variety of cancers, including tumors of the breast (9), colon (10), and gastric (11), with high expression of C5aR1 generally associated with poor prognosis (12, 13). Hu *et al.* (14) reported high expression of C5aR1, which is involved in the epithelial–mesenchymal transition in HCC cells. These studies suggest that C5aR1 may be an important anticancer drug target, and high expression of C5aR1 is expected to become a key indicator for the diagnosis and treatment of tumors.

Panton–Valentine leucocidin (PVL), a perforating toxin secreted by *Staphylococcus aureus*, has two components: LukS-PV and LukF-PV (15). It has been reported that subunit LukS-PV specifically recognizes and binds to C5aR1 (16, 17). Our previous study demonstrated that LukS-PV inhibits the proliferation of myeloid leukemia cells with high expression of C5aR1, inducing cell apoptosis and exhibiting antileukemia effects *in vivo* and *in vitro* (18, 19, 20, 21, 22). Another of our previous studies found that LukS-PV also has antitumor effects on solid tumor cells with high C5aR1 expression, including liver and lung tumors (23, 24). For example, LukS-PV was found to inhibit the migration of HCC by regulating histone deacetylase (HDAC)6 (25). These studies indicate that LukS-PV exerts anticancer activities through several mechanisms and targets, the possibility that it has other mechanisms of action deserves further study.

The Wnt/β-catenin signaling pathway is a classical pathway for regulation of embryonic development and cell proliferation (26, 27). Aberrant activation of Wnt/β-catenin signaling plays important roles in the occurrence and progression of HCC (28). In the resting state, β-catenin is phosphorylated by a degradation complex and is subsequently recognized by E3 ligase and degraded through the ubiquitination pathway (29). Binding of the Wnt ligand to receptors on the cell membrane results in dissociation of the degradation complex. β-catenin then accumulates in the cytoplasm and enters the nucleus, where it activates the transcription of downstream target genes. Nuclear accumulation of β-catenin is one of the most widely recognized markers of malignancy (27). Despite the importance of Wnt/β-catenin signaling for cancer development, the role of acetylation in its protein degradation and the molecular mechanisms involved are poorly understood.

In the current study, we demonstrated that LukS-PV targeting C5aR1 inhibited HCC cell proliferation by downregulating HDAC7 to upregulate the acetylation level of β-catenin, thus promoting the degradation of β-catenin through the ubiquitin–proteasome system. Taken together, our studies reveal a novel mechanism that LukS-PV targeting C5aR1 inhibits HCC cell proliferation by the HDAC7-Wnt/β-catenin axis, suggesting that LukS-PV may have potential as a therapeutic drug for HCC treatment.

## Results

### LukS-PV targeting C5aR1 inhibits the proliferation of HCC cells

**Supporting Figure S1:**
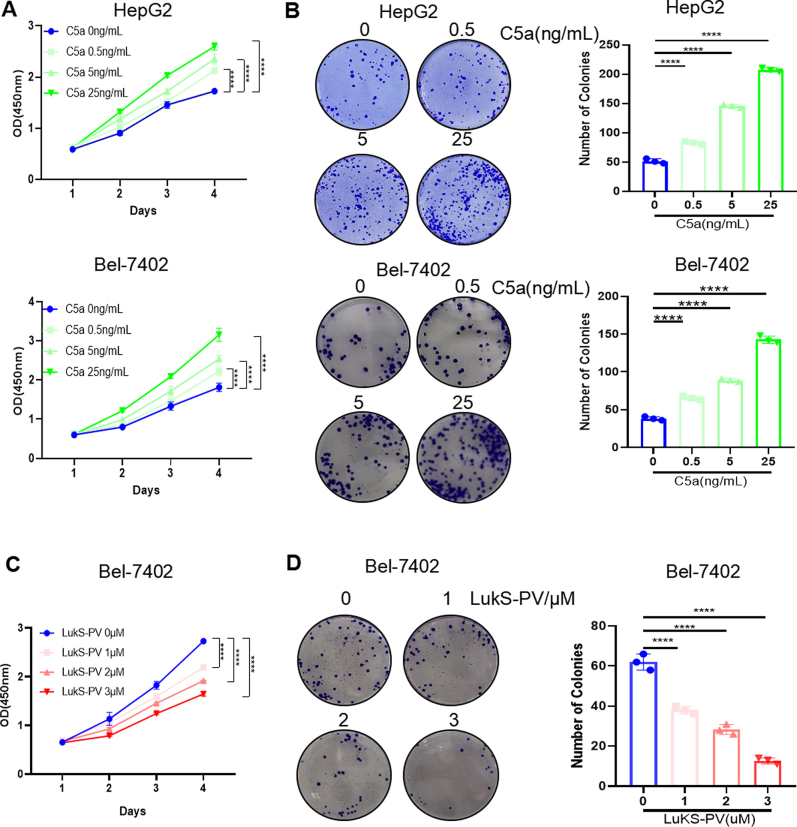


To investigate whether C5a promotes HCC cell proliferation by activating the C5a–C5aR1 axis, we treated HepG2 and Bel-7402 cells (which are highly C5aR1 expressed) with different concentrations of C5a for 4 days. The Cell Counting Kit-8 (CCK8) results showed that C5a promoted the proliferation of HCC cells in a concentration-dependent and time-dependent manner (Fig. S1*A*). Next, colony formation assays were used to further evaluate the effect of C5a on HCC cell proliferation. The results showed that C5a treatment significantly increased the number of colonies in a concentration-dependent manner compared with the vehicle control in HCC cells (Fig. S1*B*). Together, these data revealed that C5a promotes HCC cell proliferation by activating the C5a–C5aR1 axis.

**Figure 1:**
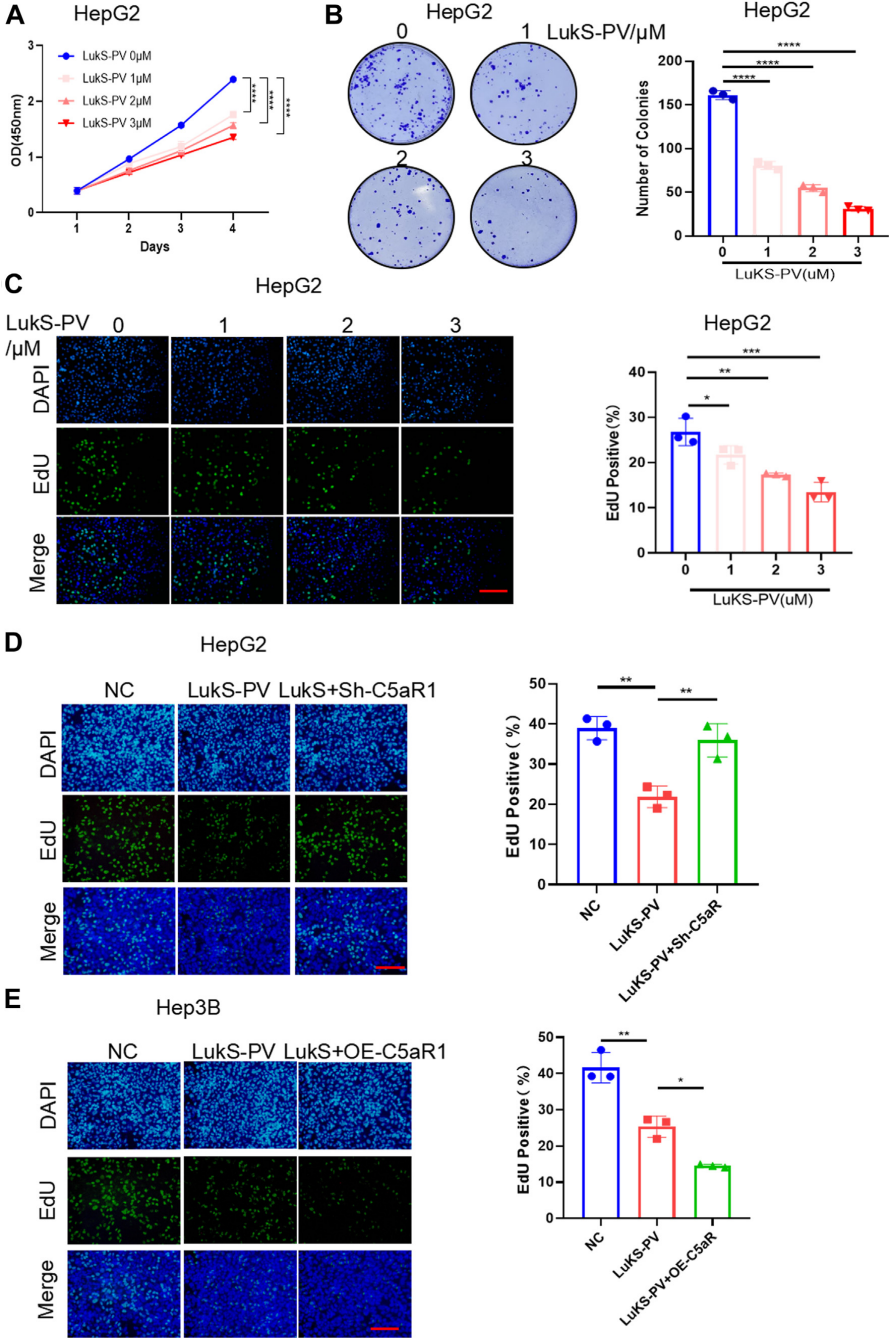
**LukS-PV targeting C5aR1 inhibits the proliferation of HCC cells.***A*, CCK-8 assay was performed to detect cell viability of HepG2 cells treated with different concentrations of LukS-PV for 1, 2, 3, and 4 days. *B*, colony formation assay was conducted to determine the clonogenic ability of HepG2 cells treated with different concentrations of LukS-PV for 14 days. *C*, EdU assays were used to analyze the viability of HepG2 cells treated with different concentrations of LukS-PV. Scale bars represent 20 μm. *D*, HepG2 cells were treated with LukS-PV or combination of LukS-PV and Sh-C5aR1. The proliferation was detected by EdU assays. Scale bars represent 20 μm. *E*, Hep3B cells were treated with LukS-PV or combination of LukS-PV and OE-C5aR1. The proliferation was detected by EdU assays. Scale bars represent 20 μm. CCK-8, Cell Counting Kit-8; HCC, hepatocellular carcinoma.

To investigate whether LukS-PV inhibits the proliferation of HCC cells by antagonizing C5a, similarly, we treated HepG2 and Bel-7402 cells with different concentrations of LukS-PV for 4 days. The CCK8 results showed that LukS-PV inhibited the proliferation of HCC cells in a concentration-dependent and time-dependent manner (Figs. 1*A*, S1*C*). Next, colony formation results also showed that LukS-PV treatment significantly reduced the number of colonies compared with the vehicle control in HCC cells (Figs. 1*B*, S1*D*). A similar effect was observed in EdU assays in HepG2 cells (Fig. 1*C*). Together, these data revealed that LukS-PV inhibits HCC cell proliferation.

To investigate whether LukS-PV inhibits the proliferation of HCC cells by targeting C5aR1, we knocked down C5aR1 expression in HepG2 cells, which are relatively high C5aR1 expression; induced the ectopic expression of C5aR1 in Hep3B cells, which are relatively low C5aR1 expression. Then, we assessed cell proliferation after knocked down or overexpressed C5aR1 and combined with LukS-PV in HCC cells. EdU results revealed that LukS-PV significantly reduced the number of EdU-positive cells, and this effect was markedly alleviated in C5aR1-knockdown cells but further enhanced in C5aR1-overexpressing cells (Fig. 1, *D* and *E*). In other words, the effect of LukS-PV on HCC cell proliferation depends on C5aR1.

### LukS-PV targeting C5aR1 inhibits HCC cell proliferation by downregulating Wnt/β-catenin signaling

**Figure 2:**
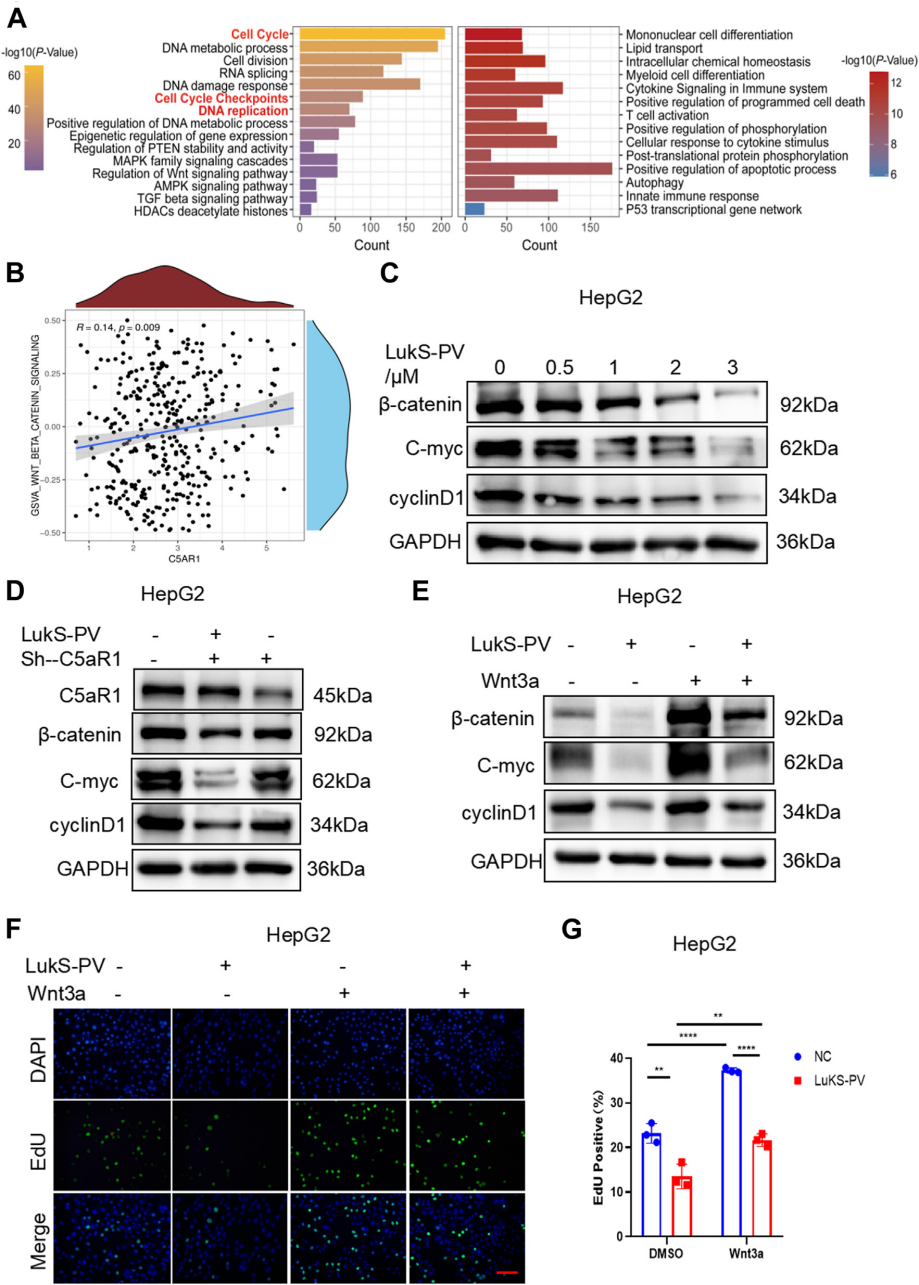
**LukS-PV targeting C5aR1 inhibits the proliferation of HCC cells by downregulating Wnt/β-catenin signaling.***A*, KEGG pathway enrichment analysis revealed the signaling pathways potentially involved in the function of LukS-PV. *B*, the correlation was found between C5aR1 and HALLMARK_WNT_ BETA_CATENIN_SIGNALING. *C*, Western blotting analysis of the levels of β-catenin, C-myc, and cyclinD1 expression in HepG2 cells treated with different concentrations of LukS-PV for 24 h. GAPDH was used as the control. *D*, HepG2 cells were treated with LukS-PV or combination of LukS-PV and Sh-C5aR1. The protein expression levels of C5aR1, β-catenin, C-myc, and cyclinD1 were detected by Western blotting. *E*, HepG2 cells pretreated with Wnt3a stimulation and then treated with LukS-PV. The protein expression levels of β-catenin, C-myc, and cyclinD1 were detected by Western blotting. *F* and *G*, EdU assay analyses of proliferation in HepG2 cells pretreated with Wnt3a stimulation and then treated with LukS-PV. Scale bars represent 20 μm. HCC, hepatocellular carcinoma; KEGG, Kyoto Encyclopedia of Genes and Genomes.

**Supporting Figure S2:**
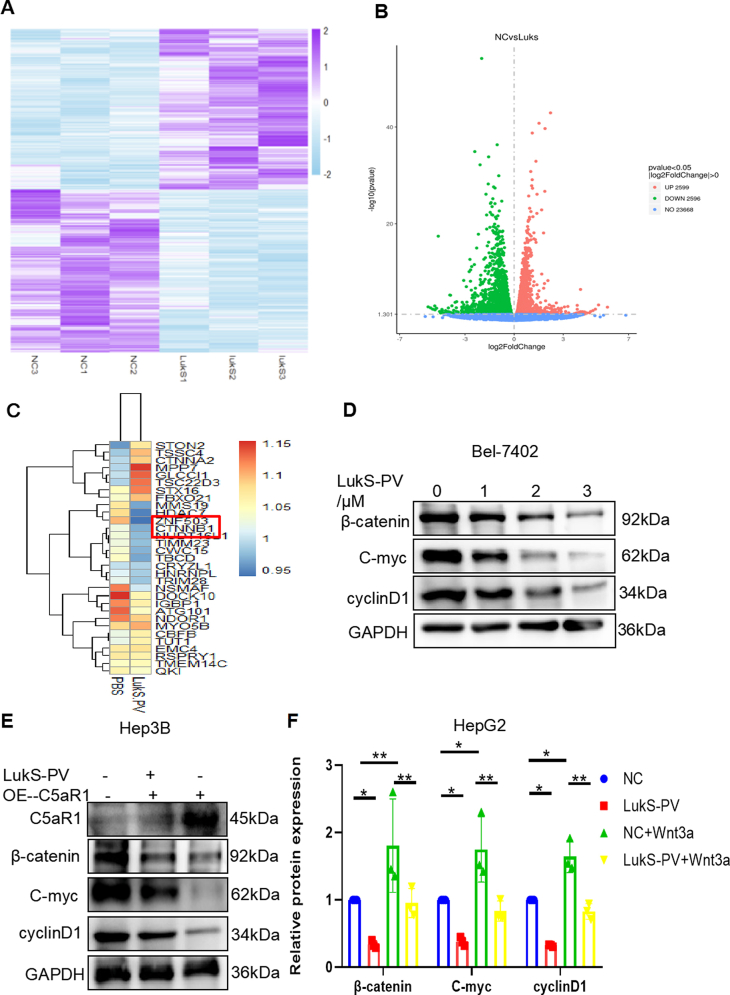


To explore the potential mechanism of LukS-PV targeting C5aR1 inhibiting the proliferation of HCC cells, we first performed RNA-Seq. Heatmap analysis revealed significant differences in genes regulated by LukS-PV treatment compared with the PBS group (Fig. S2*A*). The results indicated that LukS-PV treatment upregulated 2599 genes and downregulated 2596 genes compared with the PBS group in HepG2 cells (Fig. S2*B*). After the differentially expressed genes were subjected to Kyoto Encyclopedia of Genes and Genomes pathway analysis, the data revealed relevant enrichment in cell cycle, DNA damage response pathway, and tumor-related pathways such as mitogen-activated protein kinase and PTEN signaling pathway. Cell cycle, cell cycle checkpoints, and DNA replication pathways were involved in cell proliferation and attracted our attention (Fig. 2*A*).

In addition, we conducted a correlation analysis on the enrichment scores of C5aR1 and the HALLMARK gene set of MSigDB in The Cancer Genome Atlas (TCGA) dataset. Interestingly, a positive correlation was found between C5aR1 and HALLMARK_WNT_ BETA_CATENIN_SIGNALING (R = 0.14, *p* = 0.009) (Fig. 2*B*). The Wnt/β-catenin signaling pathway is a classical pathway for regulation of cell proliferation (26). The β-catenin is a core member of Wnt signaling pathway (27). Aberrant activation of Wnt/β-catenin signaling plays important roles in the occurrence and progression of HCC (26, 28). Meanwhile, we found that expression of *CTNNB1* was downregulated in the LukS-PV treatment group compared with PBS group by quantitative proteomics sequencing (Fig. S2*C*). So we first confirmed that LukS-PV downregulated β-catenin in a concentration-dependent manner in HepG2 and Bel-7402 cells using Western blotting (WB) (Figs. 2*C*, S2*D*). In addition, as downstream target genes of Wnt/β-catenin, C-myc and cyclinD1 were also decreased after LukS-PV treatment (Figs. 2*C*, S2*D*).

To investigate whether LukS-PV inhibits the proliferation of HCC cells through Wnt/β-catenin signaling by targeting C5aR1, similarly, we knocked down C5aR1 expression in HepG2 cells and induced the ectopic expression of C5aR1 in Hep3B cells. Then, we assessed cell proliferation after knocked down or overexpressed C5aR1 and combined with LukS-PV by WB in HCC cells. WB results revealed that LukS-PV downregulated β-catenin, C-myc, and cyclinD1, but this effect was markedly alleviated in C5aR1-knockdown cells and further enhanced in C5aR1-overexpressing cells (Figs. 2*D*, S2*E*). In other words, LukS-PV inhibiting the proliferation of HCC cells through Wnt/β-catenin signaling depends on C5aR1.

To further determine whether LukS-PV inhibited the proliferation of HCC cells through Wnt/β-catenin signaling, HCC cells were pretreated with Wnt3a (Wnt/β-catenin signaling agonist) stimulation and then treated with LukS-PV. The inhibition of proliferation in HCC cells was detected by WB. In contrast, LukS-PV treatment led to a marked decrease in HCC cell proliferation (Figs. 2*E*, S2*F*), and Wnt3a significantly attenuated the inhibitory effects of LukS-PV on HCC cell proliferation compared with LukS-PV treatment group (Figs. 2*E*, S2*F*). A similar effect was observed using EdU assay (Fig. 2, *F* and *G*). Together, these data revealed that LukS-PV targeting C5aR1 inhibits HCC cell proliferation by downregulating Wnt/β-catenin signaling.

### LukS-PV inhibits HCC cell proliferation by upregulating the acetylation of β-catenin to promote its degradation

**Figure 3:**
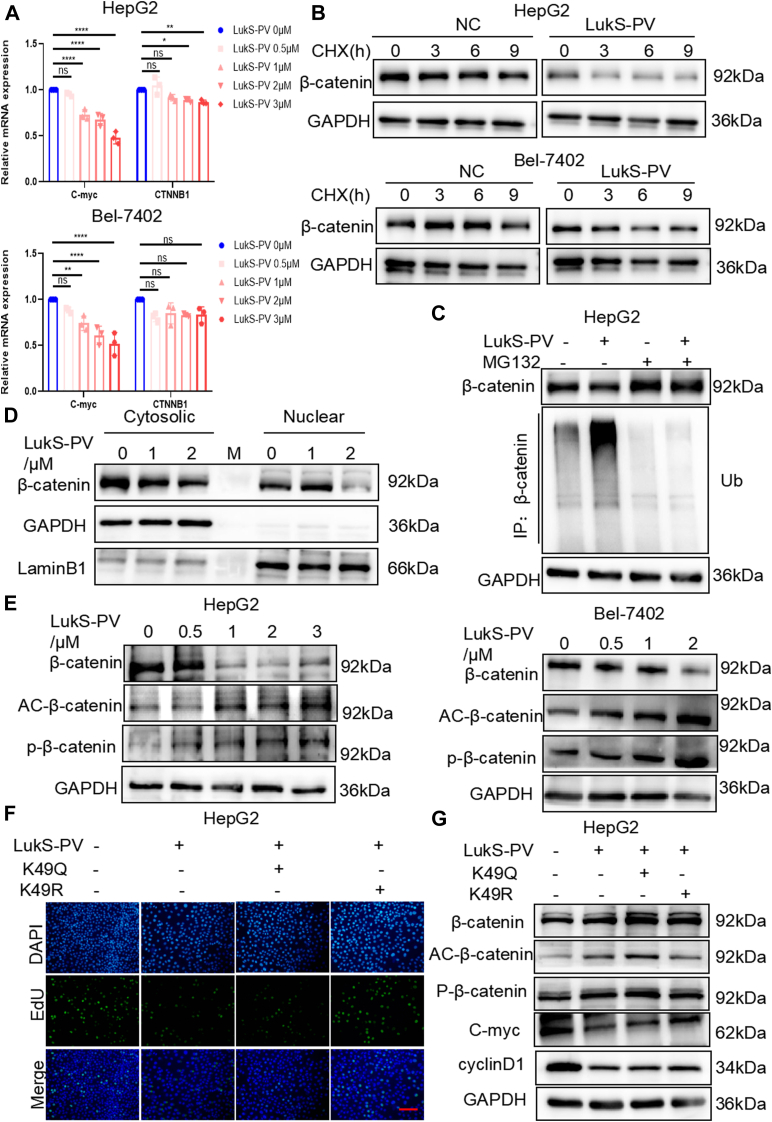
**LukS-PV inhibits HCC cell proliferation by upregulating the acetylation of β-catenin to promote its degradation.***A*, quantitative RT–PCR was performed to analyze the mRNA level of *C-myc* and *CTNNB1* in HepG2 and Bel-7402 cells treated with LukS-PV. *B*, Western blotting analysis of the levels of β-catenin in HepG2 and Bel-7402 cells treated with 20 μM CHX for the indicated times, with and without LukS-PV treatment, showing the half-life of β-catenin. *C*, Western blotting analysis of the levels of β-catenin and ubiquitinated β-catenin in HepG2 cells treated with LukS-PV and with or without the proteasome inhibitor MG132 (10 μM) for 5 h. *D*, Western blotting analysis of cytosolic and nuclear fractions of β-catenin after treated with LukS-PV in HCC cells. *E*, Western blotting analysis of acetylated and phosphorylated β-catenin in HCC cells treated with LukS-PV. *F*, EdU assay analysis of proliferation in HepG2 cells transfected with the β-catenin K49Q and K49R mutant expression plasmids and treated with LukS-PV. Scale bars represent 20 μm. *G*, Western blotting analysis of the levels of acetylated and phosphorylated β-catenin, C-myc, cyclinD1, and β-catenin in HepG2 cells transfected with the β-catenin K49Q and K49R mutant expression plasmids and treated with LukS-PV. CHX, cycloheximide; HCC, hepatocellular carcinoma.

Interestingly, quantitative RT–PCR (qRT–PCR) analysis showed that LukS-PV treatment did not induce significant changes in *CTNNB1* expression at the mRNA level in HCC cells, *C-my*c gene is a positive control here (Fig. 3*A*). These results indicated that LukS-PV might downregulate β-catenin expression through protein degradation. To test this hypothesis, we exposed vehicle control and LukS-PV-treated cells to the protein synthesis inhibitor cycloheximide and obtained protein extracts at the indicated time points. The half-life of β-catenin was shorter in LukS-PV-treated cells than in vehicle control cells (Fig. 3*B*). Next, we added the proteasome inhibitor MG-132 to HCC cells and found that the LukS-PV-mediated downregulation of β-catenin protein expression was reversed (Fig. 3*C*). In addition, LukS-PV-treated HCC cells showed increased accumulation of ubiquitinated β-catenin, and this accumulation was blocked by MG-132 treatment (Fig. 3*C*).

Hyperacetylation of β-catenin reportedly leads to phosphorylation, promoting its degradation *via* the ubiquitin–proteasome system (30). The degradation of β-catenin protein leads to a decrease in enteration of the nucleus. To determine whether LukS-PV affected the subcellular localization of β-catenin, we separated cytosolic and nuclear fractions of HCC cells after treated with LukS-PV. The WB results showed that LukS-PV treatment inhibited the nuclear import of β-catenin (Fig. 3*D*).

**Supporting Figure S3:**
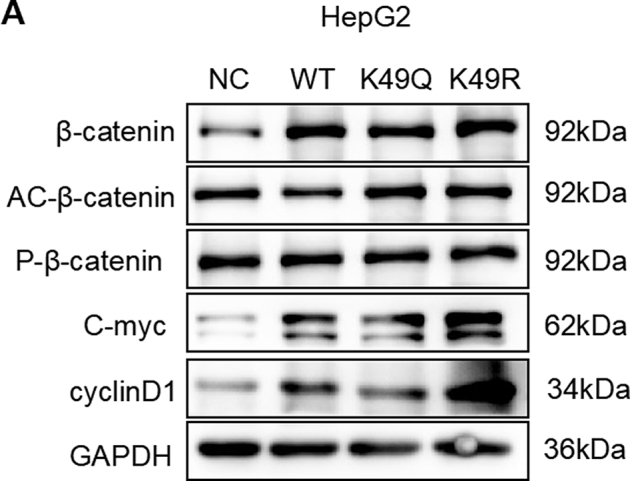


Interestingly, WB showed higher levels of acetylated and phosphorylated β-catenin in LukS-PV-treated HCC cells (Fig. 3*E*). Previous studies have found the K49 residue of β-catenin was identified as the major acetylation site (31). So we hypothesized that LukS-PV may upregulate the acetylation level of β-catenin, leading to β-catenin degradation and ultimately inhibiting the proliferation of HCC cells. To test this hypothesis, we targeted the highly conserved K49 locus for construction of a K49Q acetylation–mimicking mutant and a K49R acetylation–resistant mutant of β-catenin. WB was used to detect the expression level of related proteins after overexpressing K49Q and K49R mutants. WB results revealed that, compared with NC group, the expression of β-catenin was elevated in WT, K49Q, and K49R β-catenin groups; but the change of acetylation and phosphorylation levels of β-catenin in these groups was not obvious. The results may be due to the spatial conformation of the mimicked mutant, which is not recognized by modification antibodies at specific sites. But more importantly, compared with NC group, the expression of C-myc and cyclinD1 was elevated in WT, K49Q, and K49R β-catenin groups. Compared with WT β-catenin, the K49Q mutants downregulated the expression of C-myc and cyclinD1; in contrast, the K49R mutants upregulated the expression of C-myc and cyclinD1 (Fig. S3*A*). In other words, the acetylation-mimic mutant K49Q can alleviate β-catenin promotion of cell proliferation, and the acetylation-resistant mutant K49R can further enhance β-catenin promotion of proliferation. We further added LukS-PV to treat these cells and detected the effect on cell proliferation. EdU assays showed that the K49Q mutation enhanced the inhibitory effect of LukS-PV on proliferation, whereas the K47R mutation reversed it (Fig. 3*F*). A similar effect was observed in WB assays (Fig. 3*G*). But no effect was observed on expression of cyclinD1 upon overexpression of K49Q and K49R combined with LukS-PV; it is suggested that there are other ways of regulating cyclinD1 by LukS-PV, in addition to the regulation through β-catenin acetylation. Taken together, these data indicated that LukS-PV upregulates the acetylation of β-catenin to promote its protein degradation and thereby inhibit HCC cell proliferation.

### HDAC7 mediates the deacetylation of β-catenin to inhibit its protein degradation and promote HCC cell proliferation

**Figure 4:**
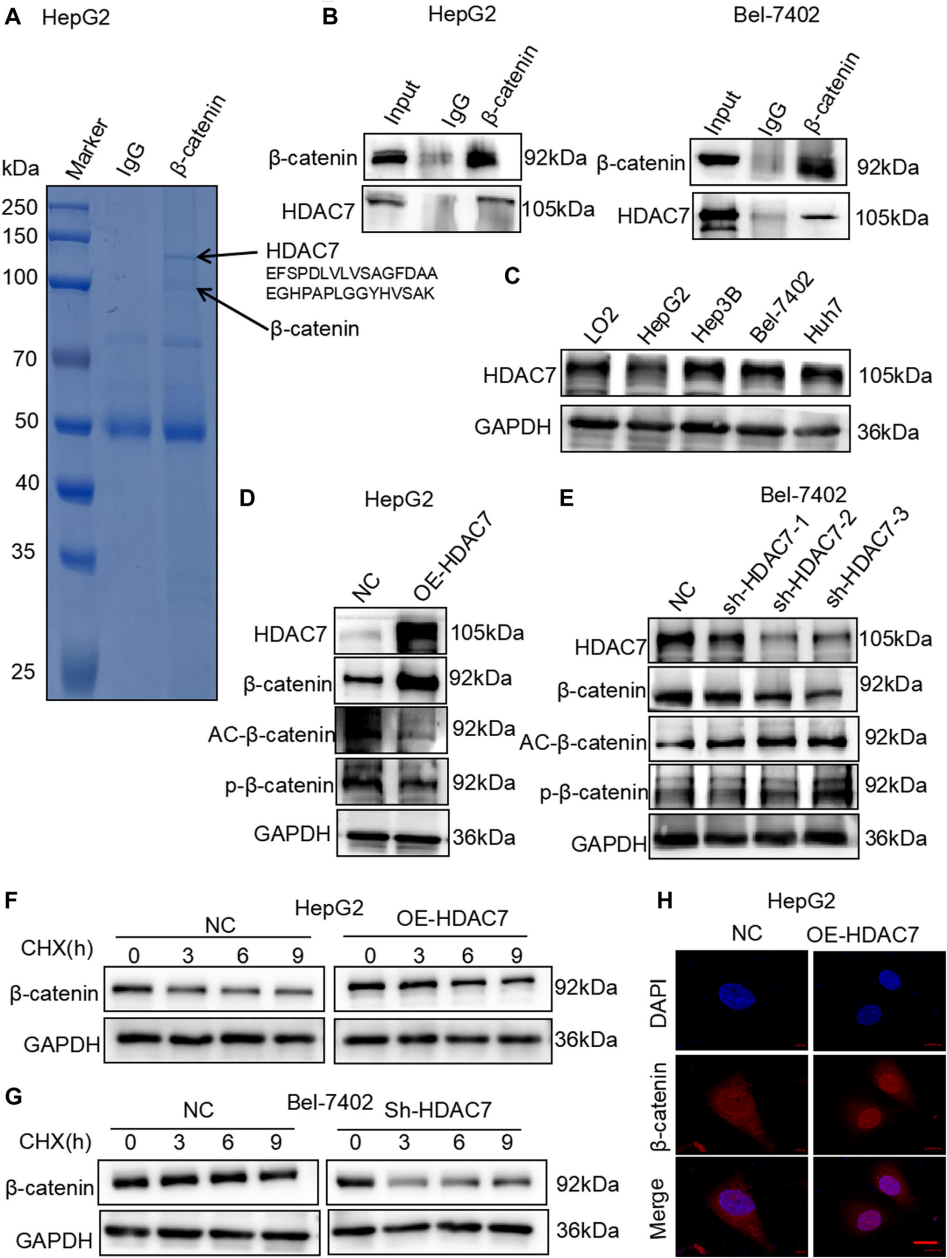
**HDAC7 mediates the deacetylation of β-catenin and inhibits its degradation in HCC cells.***A*, mass spectrometry detection of an HDAC7 peptide sequence associated with affinity-purified β-catenin from HepG2 cells stably expressing β-catenin. The peptide (EFSPDLVLVSAGFDAAEGHPAPLGGYHVSAK) showing in this panel is the HDAC7 sequence detected by mass spectrometry. *B*, Western blotting verification IP of β-catenin and HDAC7 in HepG2 and Bel-7402 cells. *C*, Western blotting analysis of the level of HDAC7 in LO2, HepG2, Hep3B, Bel-7402, and Huh7 cells. *D* and *E*, Western blotting analysis of the levels of β-catenin, acetylated and phosphorylated β-catenin in HepG2 cells with OE-HDAC7 (*D*) and Bel-7402 cells under sh-HDAC7 (*E*). *F* and *G*, Western blotting analysis of the half-life on β-catenin in HepG2 cells with OE-HDAC7 (*F*) and Bel-7402 cells with sh-HDAC7 (*G*). *H*, immunofluorescence analysis of β-catenin protein in HepG2 cells with OE-HDAC7, showing its translocation from cytoplasm to nucleus. Scale bars represent 10 μm. HCC, hepatocellular carcinoma; HDAC, histone deacetylase; IP, immunoprecipitation; OE-HDAC7, HDAC7 overexpression.

To identify the upstream histone acetylation modification enzymes involved in regulating β-catenin acetylation in HCC cells, immunoprecipitation (IP) and LC–MS/MS analyses were used to examine potential interacting proteins in HepG2 cells stably expressing β-catenin (Table S1). As shown in Figure 4*A*, affinity purification showed that HDAC7 was associated with β-catenin, which was validated by immunopurification in HepG2 and Bel-7402 cells (Fig. 4*B*). And HDAC7 is the only acetylation modification enzyme detected by mass spectrometry (MS) analyses. Studies have reported that HDAC7 can control endothelial cell growth and chondrocyte proliferation through modulation of β-catenin (32, 33). These results indicated that HDAC7 may act upstream of β-catenin to downregulate the acetylation level of β-catenin. To test this hypothesis, we first examined HDAC7 expression in HCC cell lines and the normal hepatocyte cell line L02 (Fig. 4*C*). Meanwhile, we constructed an ectopic HDAC7 overexpression (OE-HDAC7) in HepG2 cells, which exhibits relatively low HDAC7 expression compared with other HCC cell lines (Fig. 4*C*). We found that OE-HDAC7 promoted β-catenin expression while downregulating the levels of β-catenin acetylation and phosphorylation (Fig. 4*D*). In addition, we tested shRNA-mediated knockdown of HDAC7 expression in Bel-7402 cells, which exhibits relatively high HDAC7 expression compared with other HCC cell lines (Fig. 4*C*). The results showed that HDAC7 knockdown significantly reduced β-catenin while upregulating the levels of β-catenin acetylation and phosphorylation (Fig. 4*E*). More importantly, cycloheximide experiment indicated that the half-life of β-catenin was considerably extended by OE-HDAC7 but was considerably shortened by HDAC7 knockdown (Fig. 4, *F* and *G*). Furthermore, immunofluorescence assays revealed that nuclear β-catenin expression was significantly increased in OE-HDAC7 cells (Fig. 4*H*). Taken together, these data suggested that HDAC7 mediates the deacetylation of β-catenin to inhibit its protein degradation in HCC cells.

**Figure 5:**
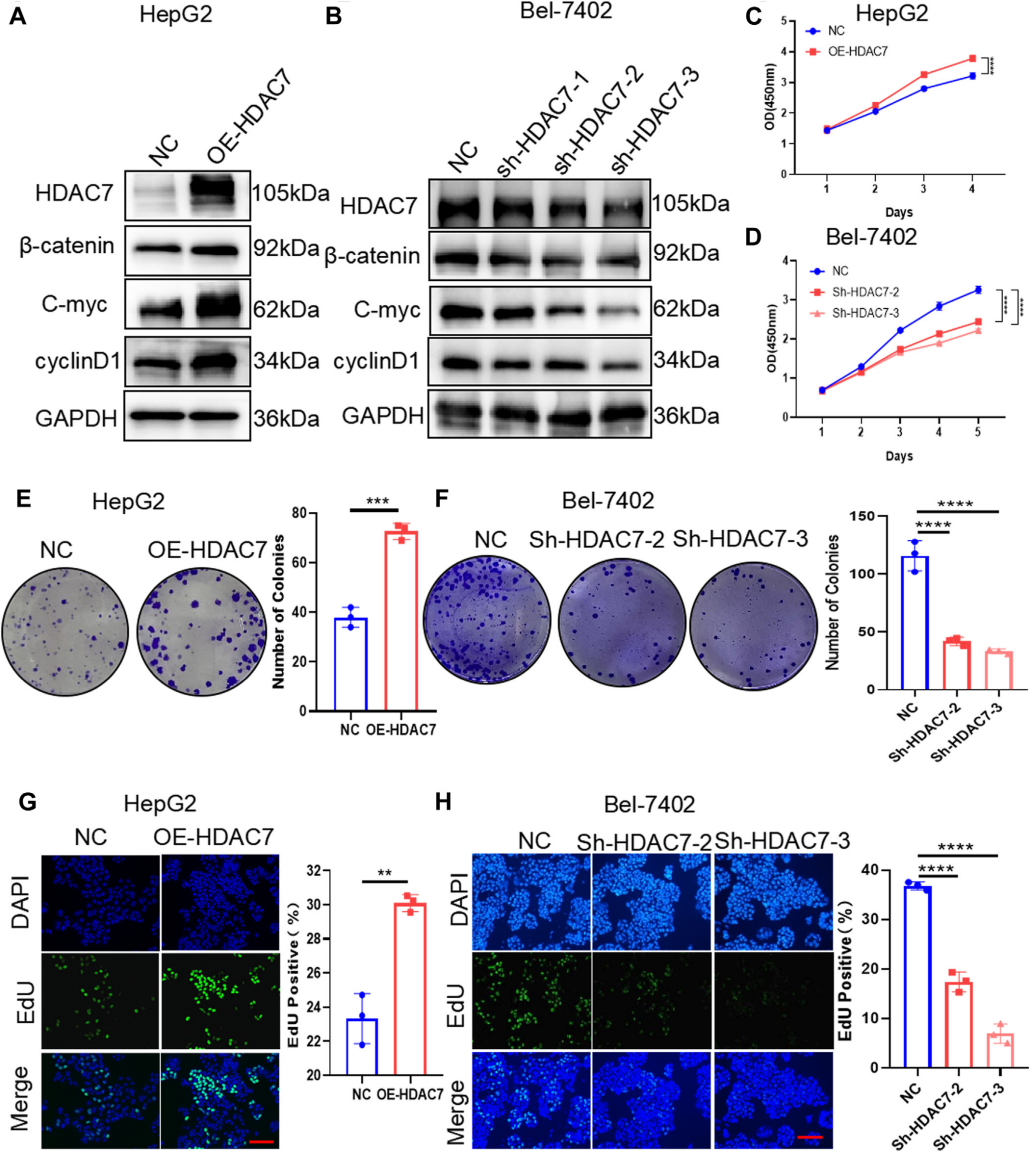
**HDAC7 promotes HCC cell proliferation.***A* and *B*, Western blotting analysis of β-catenin, C-myc, and cyclinD1 in HepG2 cells with OE-HDAC7 (*A*) and Bel-7402 cells with sh-HDAC7 (*B*). *C* and *D*, CCK-8 viability assay analysis in HepG2 cells with OE-HDAC7 (*C*) and Bel-7402 cells with sh-HDAC7 (*D*). *E* and *F*, colony formation assay analysis of viability in HepG2 cells with OE-HDAC7 (*E*) and Bel-7402 cells with sh-HDAC7 (*F*). *G* and *H*, EdU assay analysis of proliferation in HepG2 cells with OE-HDAC7 (*G*) and Bel-7402 cells with sh-HDAC7 (*H*). Scale bars represent 20 μm. CCK-8, Cell Counting Kit-8; HCC, hepatocellular carcinoma; HDAC, histone deacetylase; OE-HDAC7, HDAC7 overexpression.

**Supporting Figure S4:**
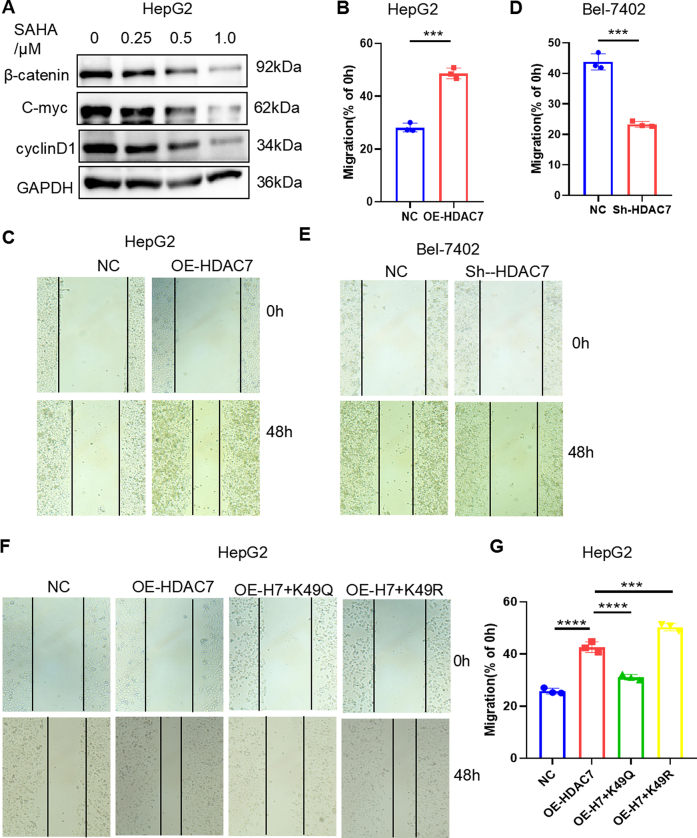


Next, we tested whether HDAC7-mediated deacetylation of β-catenin and inhibition of its protein degradation promote cell proliferation in HCC cells. WB results showed that OE-HDAC7 significantly upregulated the expression of β-catenin, C-myc, and cyclinD1 in HepG2 cells (Fig. 5*A*), whereas HDAC7 knockdown markedly downregulated the expression of these proteins in Bel-7402 cells (Fig. 5*B*). Consistently, CCK8 results showed that OE-HDAC7 significantly promoted the proliferation of HepG2 cells, whereas HDAC7 knockdown markedly inhibited the proliferation of Bel-7402 cells (Fig. 5, *C* and *D*). Similar effects were observed in colony formation and EdU assays (Fig. 5, *E*–*H*). In addition, we examined the effect of HDAC inhibitor on the expression of β-catenin by WB. The results showed that suberoylanilide hydroxamic acid (HDAC inhibitor) also can downregulate the expression of β-catenin, C-myc, and cyclinD1 in HepG2 cells. The results proved that catalytic activity of HDAC7 is essential (Fig. S4*A*).

Furthermore, studies suggested that β-catenin pathway also affects cell migration in addition to cell proliferation (34, 35). We also found that HDAC6 has essential role in cell migration of HCC cells in our previous study (25). To investigate whether HDAC7-mediated regulation of β-catenin has major impact of the migration of HCC cells, scratch experiments were performed to test cell migration after overexpression of HDAC7 in HepG2 cells and knockdown of HDAC7 in Bel-7402 cells. The results showed that HDAC7 overexpression significantly increased the migration ability of these cells, and knockdown of HDAC7 significantly decreased cell migration (Fig. S4, *B*–*E*). Furthermore, the K49Q mutation reduced HDAC7-mediated HCC cell migration, whereas the K49R mutation increased HDAC7-mediated migration in these cells (Fig. S4, *F* and *G*). Taken together, these data suggested that HDAC7 mediates the deacetylation of β-catenin to inhibit its protein degradation and promote HCC cell proliferation and cell migration.

### LukS-PV downregulates the high expression of HDAC7 by targeting C5aR1 in HCC cells

**Figure 6:**
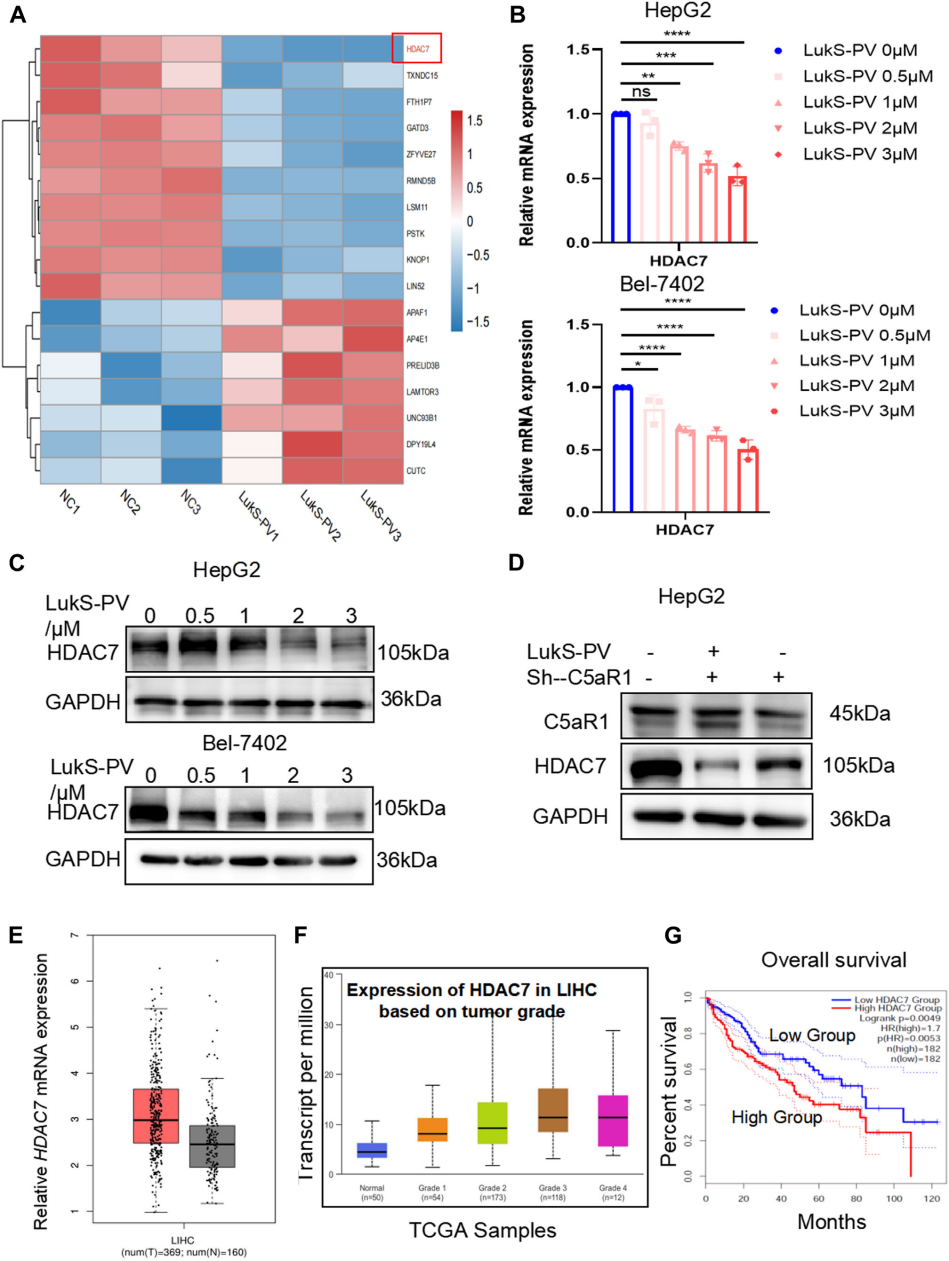
**LukS-PV downregulates the high expression of HDAC7 by targeting C5aR1 in HCC cells.***A*, comparative RNA-Seq analysis of changes in *HDAC7* mRNA expression levels in HepG2 cells treated with LukS-PV. *B* and *C*, quantitative RT–PCR (*B*) and Western blotting (*C*) analysis of HDAC7 protein and mRNA expression levels in HCC cells treated with LukS-PV. *D*, HepG2 cells were treated with LukS-PV or combination of LukS-PV and Sh-C5aR1. The protein expression levels of C5aR1 and HDAC7 were detected by Western blotting. *E*–*G*, TCGA-based analyses of correlations between HDAC7 and the occurrence of liver cancer (*E*), HDAC7 and the clinicopathological stages of liver cancer (*F*), and HDAC7 and the prognosis of liver cancer (*G*). HCC, hepatocellular carcinoma. HDAC, histone deacetylase; PV, Panton–Valentine; TCGA, The Cancer Genome Atlas.

**Supporting Figure S5:**
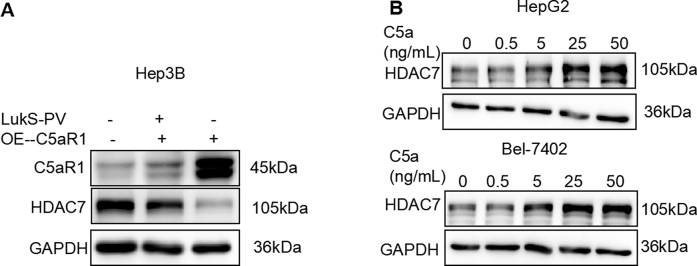


To investigate whether HDAC7 as a potential molecular target by LukS-PV inhibits HCC cell proliferation, we performed RNA-Seq of HepG2 cells treated with LukS-PV or PBS. Interestingly, we found that *HDAC7* expression was downregulated in the LukS-PV group (Fig. 6*A*). qRT–PCR and WB analyses confirmed that LukS-PV significantly downregulated HDAC7 at both the mRNA and protein levels in a concentration-dependent manner in HepG2 and Bel-7402 cells (Fig. 6, *B* and *C*). In addition, WB results revealed that LukS-PV downregulated HDAC7, but this effect was markedly alleviated in C5aR1-knockdown cells and further enhanced in C5aR1-overexpressing cells (Figs. 6*D*, S5*A*). And C5a-treated HCC cells showed that the expression of HDAC7 was upregulated (Fig. S5*B*).

To further investigate the role of HDAC7 in liver cancer, we used GEPIA to analyze *HDAC7* expression in liver cancer cases from TCGA. These data showed that *HDAC7* expression is dramatically increased in tumor samples compared with normal tissues (Fig. 6*E*) and is closely associated with the clinical and pathological stages of liver cancer (Fig. 6*F*). Kaplan–Meier survival analysis showed that patients with high *HDAC7* expression also had reduced overall survival (Fig. 6*G*). Taken together, these results indicated that HDAC7 as a potential prognostic marker for liver cancer, and LukS-PV can downregulate the high expression of HDAC7 by targeting C5aR1 in HCC cells.

### LukS-PV inhibits HCC cell proliferation by downregulating HDAC7-Wnt/β-catenin signaling

**Figure 7:**
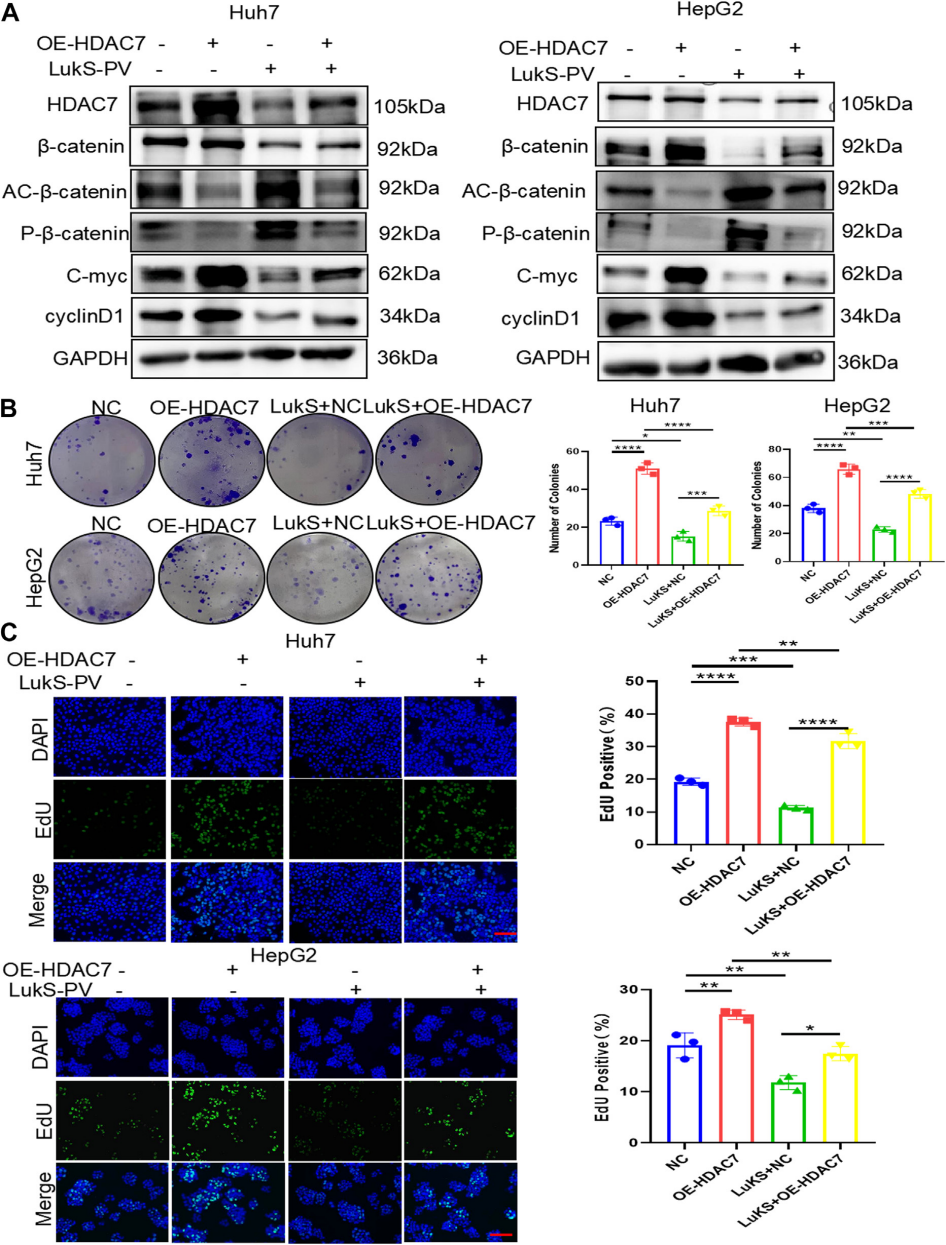
**LukS-PV inhibits HCC cell proliferation by downregulating HDAC7-Wnt/β-catenin signaling.***A*, Western blotting analysis of related protein expression in HCC cells with OE-HDAC7 and LukS-PV or combination of LukS-PV and OE-HDAC7. *B* and *C*, colony forming assay analysis of viability (*B*) and EdU assay analysis of proliferation (*C*) in HCC cells with OE-HDAC7 and LukS-PV or combination of LukS-PV and Sh-C5aR1. Scale bars represent 20 μm. HCC, hepatocellular carcinoma; HDAC, histone deacetylase; OE-HDAC7, HDAC7 overexpression; PV, Panton–Valentine.

On the basis of the aforementioned studies, we hypothesized that LukS-PV can inhibit HCC cell proliferation by downregulating HDAC7–Wnt/β-catenin signaling. To verify this hypothesis, Huh-7 and HepG2 cells were selected to perform overexpression and rescue experiments, based on the naturally low expression levels of HDAC7 in HCC cell lines. WB results showed that OE-HDAC7 significantly increased the expression levels of β-catenin, C-myc, and cyclinD1 but reduced the acetylation and phosphorylation levels of β-catenin (Fig. 7*A*). Conversely, treatment with LukS-PV without OE-HDAC7 expression greatly decreased the expression levels of β-catenin, C-myc, and cyclinD1 but enhanced the acetylation and phosphorylation levels of β-catenin. In addition, OE-HDAC7 significantly impaired the inhibitory effects of treatment with LukS-PV (Fig. 7*A*). Colony formation results showed that LukS-PV treatment group significantly reduced the number of colonies, and OE-HDAC7 group significantly increased the number of colonies. Furthermore, OE-HDAC7 impaired the inhibitory effect of LukS-PV treatment on HCC cell proliferation (Fig. 7*B*). Similar effects were observed in colony formation and EdU assays (Fig. 7*C*).

**Figure 8:**
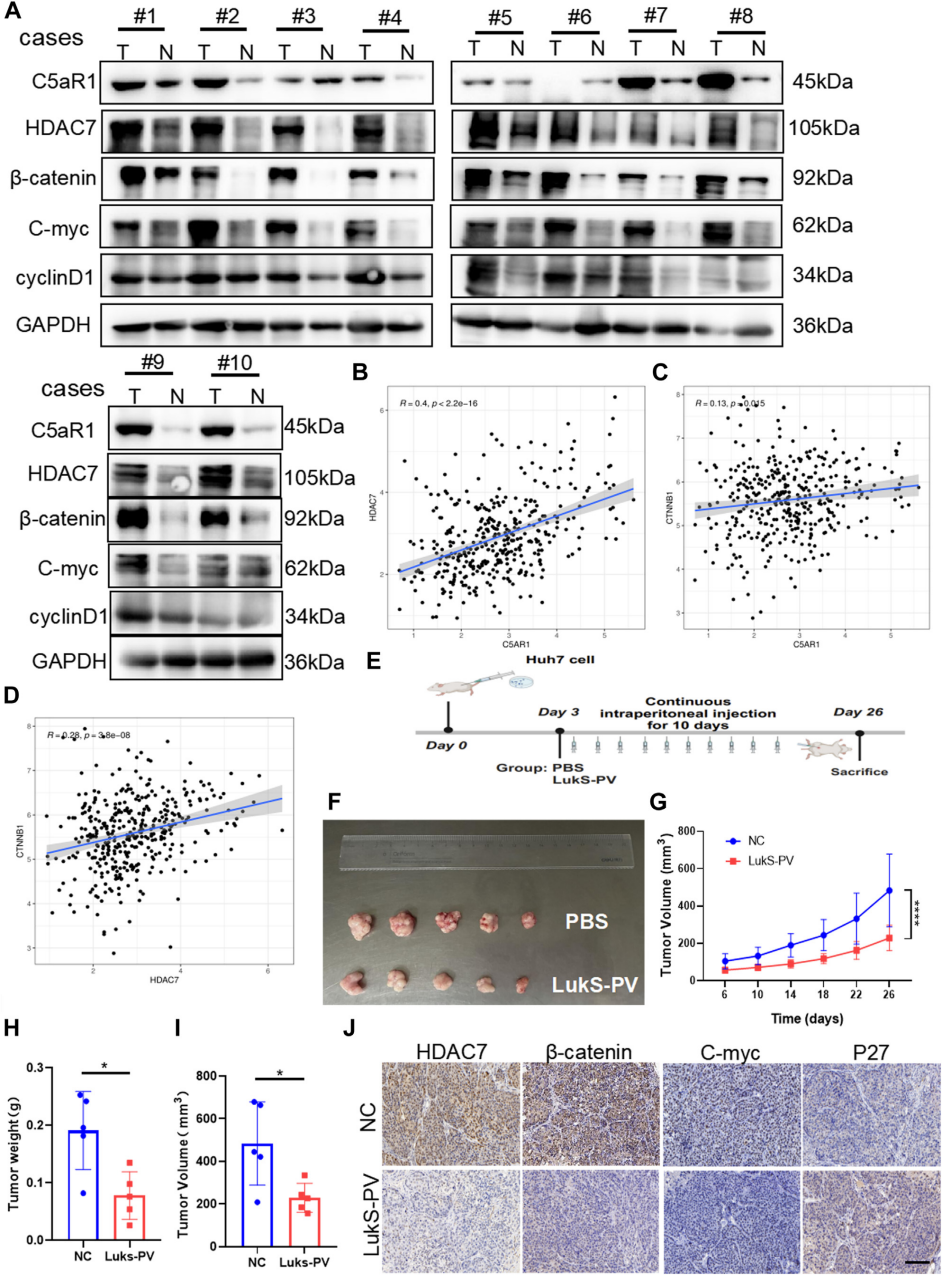
**LukS-PV inhibits HCC cell proliferation by downregulating HDAC7-Wnt/β-catenin signaling.***A*, Western blotting analysis of related protein expression in 10 HCC tissues compared with the adjacent tissues. *B*–*D*, analysis of the correlation between the expression levels of C5aR1 and HDAC7 (*B*), C5aR1 and CTNNB1 (*C*), and HDAC7 and CTNNB1 (*D*) in liver cancer cases from TCGA database. *E*, schematic of the mouse xenograft model treated with PBS and LukS-PV after subjecting Huh7 cells. *F*, the size of tumors separated from nude mice in PBS group and LukS-PV group. *G*–*I*, tumor growth curves (*G*), tumor weight (*H*), and tumor volume (*I*) were measured and calculated in PBS group and LukS-PV group. *J*, representative images of immunohistochemistry staining of HDAC7, β-catenin, C-myc, and P27 in tumor tissues from xenograft model mice. Scale bars represent 50 μm. HCC, hepatocellular carcinoma; HDAC, histone deacetylase; TCGA, The Cancer Genome Atlas.

To further explore the correlation between HDAC7 and Wnt/β-catenin signaling *in vivo*, we determined the levels of associated proteins in 10 HCC tissues by WB. The results showed that the levels of C5aR1, HDAC7, β-catenin, C-myc, and cyclinD1 were significantly increased in HCC tissues compared with the adjacent tissues (Fig. 8*A*). Next, we investigated the relationship between the expression levels of C5aR1, HDAC7, and CTNNB1 in liver cancer cases from TCGA database. In accordance, the results revealed a positive association between the expression of C5aR1 and HDAC7 (R = 0.4, *p* < 0.05), C5aR1 and *CTNNB1* (R = 0.13, *p* = 0.015), and HDAC7 and CTNNB1 (R = 0.28, *p* < 0.05) (Fig. 8, *B*–*D*).

In addition, to determine whether LukS-PV inhibited liver cancer progression by downregulating HDAC7–Wnt/β-catenin signaling *in vivo*. Using a xenograft mouse model (Fig. 8*E*), we found that LukS-PV inhibited tumor growth in nude mice (Fig. 8, *F*–*I*). In addition, immunohistochemistry showed that LukS-PV decreased the expression of C-myc, HDAC7, and β-catenin, increased the expression of P27 compared with the PBS control group (Fig. 8*J*). Taken together, these results indicated that LukS-PV has the ability to inhibit the proliferation of HCC cells by downregulating HDAC7–Wnt/β-catenin signaling *in vitro* and *in vivo*.

**Figure 9:**
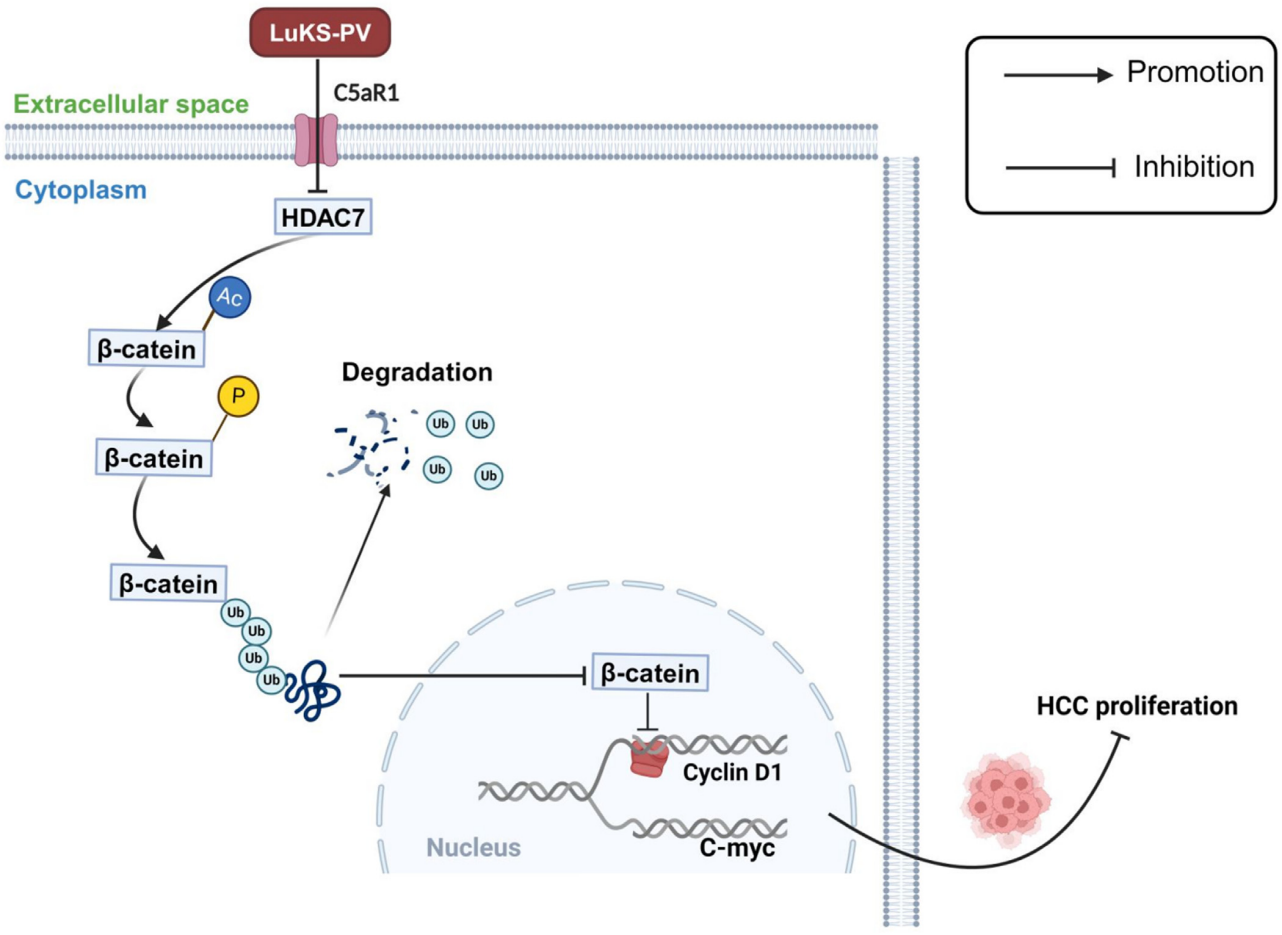
**Schematic representation of the study.** Proposed mechanism of LukS-PV inhibits HCC cell proliferation by downregulating HDAC7–Wnt/β-catenin signaling. HCC, hepatocellular carcinoma; HDAC, histone deacetylase.

Overall, our findings indicated that LukS-PV targeting C5aR1 downregulated HDAC7 to upregulate the acetylation level of β-catenin, thus promoted its protein degradation and reduced entry into the nucleus, led to target gene transcription inactivated, and ultimately inhibited HCC cell proliferation (Fig. 9).

## Discussion

In recent years, studies have found that molecular targeted therapy has unique advantages in controlling HCC malignant proliferation and metastasis (2, 30, 36, 37). The complement system, which can change the biological behavior of cancer cells, has recently become a new molecular target of cancer research (7, 8, 13). Our previous studies found that the bacterial toxin LukS-PV induced differentiation and apoptosis in leukemia cells by targeting the C5aR1 (18, 19, 20, 21). In this study, we demonstrated that LukS-PV targeting C5aR1 inhibits HCC cell proliferation by the HDAC7-Wnt/β-catenin axis, suggesting the strong potential of LukS-PV as a therapeutic cancer drug for HCC treatment.

One way the complement system affects the growth of tumors is by regulating the immune response (13). C5aR1 is a member of the G protein–coupled receptor family, which share conserved seven-transmembrane helical stereo structures. As the endogenous ligand of C5aR1, C5a binds to C5aR1 to regulate the immune response *via* the classical complement activation pathway (38). Furthermore, it has also been reported that the C5a–C5aR1 axis promotes the proliferation and metastasis of tumor cells by reducing the apoptosis of neutrophils and promoting an immunosuppressive microenvironment (7, 8). In 2021, the US Food and Drug Administration approved the C5aR1 inhibitor avacopan for the treatment of severe active antineutrophil cytoplasmic autoantibody–associated vasculitis (39). Given the apparent importance of C5aR1 in the occurrence and development of a variety of tumors, it is expected to become a molecular target for the diagnosis and treatment of tumors with high expression of C5aR1. This study highlights the potential application of LukS-PV in tumor therapy and facilitates the development of antitumor agents targeting complement receptor C5aR1.

Bacterial toxins exhibiting target specificity and cytotoxicity also represent a new hotspot for the development of novel antitumor drugs (40). PVL, a two-component pore-forming cytosolic toxin secreted by *S. aureus*, was discovered by Van de Velde and isolated from hemolysin by Panton and Valentine in 1932 (41). The LukS-PV subunit of PVL reportedly targets C5aR1 on cell membranes (17). Our previous study demonstrated that LukS-PV can induce differentiation and apoptosis in leukemia cells by targeting C5aR1, displaying antileukemia activity *in vitro* and *in vivo*, without noticeable side effects in mice (18, 19, 20, 21). Furthermore, as C5aR1 was highly expressed in HCC cells, our previous study further found that LukS-PV inhibited HCC migration by downregulating HDAC6 expression (25). These investigations indicated that LukS-PV exerts anticancer activities through multiple mechanisms and targets.

Abnormal activation of the WNT/β-catenin signaling pathway can affect cell proliferation and migration, playing an important role in tumors (42). Nuclear accumulation of β-catenin is one of the most widely recognized markers of malignancy (43). Despite the importance of Wnt/β-catenin signaling for development, tissue renewal, and cancer, the mechanisms by which β-catenin enters and is retained in the nucleus under Wnt stimulation are poorly understood. Recent studies have shown that C5a–C5aR1 can enhance β-catenin stability by assembling a complex with KCTD5/cullin3/Roc-1 and β-catenin to promote the switch of polyubiquitination of β-catenin from K48 to K63 (44). A role for acetylation has been implicated by studies in which overexpressed acetylases p300 or CBP were found to interact with the C terminus of β-catenin, leading to lysine acetylation, nuclear entry of β-catenin, and increased oncogenic potential (45, 46). However, the possible effects of mutation of the acetylated residue on β-catenin protein degradation and the molecular mechanisms involved are poorly understood.

HDAC7 is reportedly highly expressed and play cancer-promoting roles in tumor cells, such as breast and lung; however, there have been few studies of HDACs in liver cancer (47, 48, 49). One study by Kim *et al.* (50) reported that HDAC7 was highly expressed in liver cancer and was negatively correlated with clinical prognosis. In this study, we found that HDAC7 plays a biological role in promoting HCC cell proliferation. Our results support the strategy of developing anti-HCC therapies that inhibit HDAC7. HDAC7 can exert oncogenic functions by regulating the acetylation of histone and nonhistone proteins. Caslini (49) reported that HDAC7 promotes the growth and metastasis of breast cancer by downregulating the acetylation of H3K27. Lei et al. (47) reported that HDAC7 inhibits STAT3 transcription by mediating its deacetylation to promote the occurrence of lung cancer. Studies have reported that HDAC7 can control endothelial cell growth and chondrocyte proliferation through modulation of β-catenin (32, 33). However, the effect of HDAC7 on the acetylation of β-catenin in liver cancer had not been reported. This study verified the binding of HDAC7 to β-catenin and demonstrated that HDAC7 regulates the deacetylation level of β-catenin in HCC. Furthermore, we demonstrated that β-catenin is a novel substrate of HDAC7.

There are some limitations in this study. First, we demonstrated that LukS-PV inhibited HCC proliferation by downregulating Wnt/β-catenin signaling pathway. However, the effect of acetylation of β-catenin appears to be controversial as for protein stabilization and transcriptional activation. In addition to the expression of Wnt/β-catenin signaling, the change in the β-catenin transcriptional activity by acetylation should be analyzed using the TCF/LEF reporter in future experiments. Second, we found that LukS-PV upregulated the acetylation level of β-catenin by HDAC7, then upregulating the phosphorylation level of β-catenin, thus promoting the degradation of β-catenin through the ubiquitin-proteasome system. It has been reported that HDAC7 can deacetylate β-catenin to affect its phosphorylation and lead to its ubiquitination degradation in glioma (31). HDAC6 also can deacetylate β-catenin at lysine 49 and inhibit β-catenin phosphorylation at serine 45, promote epidermal growth factor–induced β-catenin nuclear localization, and lead to tumor cell proliferation (51). But the crosstalk between acetylation, phosphorylation, and ubiquitination of β-catenin by LukS-PV and HDAC7 are need to further exploration.

In this study, we found that bacterial toxin LukS-PV targeting complement receptor C5aR1 downregulated HDAC7 expression to upregulate the acetylation level of β-catenin, thus promoting the degradation of β-catenin and reducing entry into the nucleus, led to target gene transcription inactivation, and ultimately inhibited HCC cell proliferation. Together, these findings reveal a novel mechanism that LukS-PV as a bacterial toxin to inhibit HCC cell proliferation through epigenetic remodeling by targeting complement receptor C5aR1, laying the foundation of LukS-PV as a promising candidate for HCC treatment.

## Experimental procedures

### Cell culture

The LO2, HepG2, Bel-7402, Hep3B, and Huh-7 human cell lines were purchased from Shanghai Cell Bank of Chinese Academy of Sciences. LO2 and Bel-7402 cells were cultured in RPMI1640 (Gibco) supplemented with 10% fetal bovine serum, 100 U/ml penicillin, and 100 mg/ml streptomycin. HepG2, Hep3B, and Huh-7 cells were cultured in Dulbecco's modified Eagle's medium (Gibco) supplemented with 10% fetal bovine serum, 100 U/ml penicillin, and 100 mg/ml streptomycin. All cells were cultured at 37 °C with 5% CO_2_ in a humidified incubator.

### Patients and specimens

HCC tissues and matched adjacent normal tissues were collected from February 2023 to March 2024 at the First Afﬁliated Hospital of USTC (USTC). None of these patients had received interventional therapy, chemotherapy, or radiotherapy before partial hepatectomy. Immediately after removal, tumor tissues and adjacent normal liver tissues were frozen in liquid nitrogen or ﬁxed with 4% paraformaldehyde. This study was approved by the Ethics Committee of the First Afﬁliated Hospital of USTC. This work abide by the Declaration of Helsinki principles.

### Production and purification of recombinant LukS-PV

The LukS-PV sequence was amplified from PVL-positive *S. aureus* isolates by PCR. PCR products were digested with XhoI (catalog no.: R6161; Promega) and BamHI (catalog no.: R6021; Promega) and ligated into the pET28a vector. Recombinant LukS-PV was generated as described previously (29). This procedure produced six recombinant His-tagged LukS-PV proteins, which were purified using the His-Bind Purification Kit (catalog no.: 70239-M; Millipore) in accordance with the manufacturer’s protocol.

### RNA-Seq

Total RNA from HepG2 cells treated with LukS-PV or PBS was extracted using the RNeasy Mini Kit following the manufacturer’s protocol. Paired-end libraries were synthesized with the TruSeq RNA Sample Preparation Kit. Briefly, the poly(A)-containing mRNA molecules were purified using oligo (dT) attached to magnetic beads. Library construction and sequencing were performed at Shanghai Sinomics Corporation.

### RNA extraction and real-time PCR

Total RNA was extracted from cultured cells using TRIzol reagent and reverse-transcribed into complementary DNA using a PrimeScript RT reagent kit, in accordance with the manufacturer’s protocol. Real-time PCR was performed on a Thermo Fisher Scientific QuantStudio5 Real-Time PCR instrument using SYBR Green Master Mix. The qRT–PCR primers used are listed in Table S3. All samples were normalized to *GAPDH* as the internal control, and relative mRNA expression levels were calculated using the 2^−ΔΔCt^ method. All experiments were repeated three times.

### Transfection

β-catenin, HDAC7, and C5aR1 expression plasmids and shRNA against HDAC7 and C5aR1 were purchased from Gene Pharma. HCC cells were transfected with plasmid or shRNA using Lipofectamine 2000, in accordance with the manufacturer’s protocol. The primer sequences used to amplify the indicated genes for cloning into expression plasmids are listed in Table S3.

### WB and ubiquitination analysis

Cellular proteins were extracted from cells using radioimmunoprecipitation assay buffer. Protein concentrations were determined using a BCA Protein Assay Kit. Each sample of 30 μg protein was denatured in 5× SDS loading buffer at 100 °C for 10 min, separated by SDS-PAGE, and transferred to 0.45-μm nitrocellulose membranes (catalog no.: HATF00010; Millipore). Membranes were blocked with 5% nonfat milk for 1 h and incubated overnight at 4 °C with specific primary antibodies at the dilutions specified by the manufacturer. The membranes were then washed and incubated for 1 h with secondary antibodies. The bands were detected with an enhanced chemiluminescence kit (catalog no.: SQ201; Epizyme). For β-catenin ubiquitination assays, the cells were treated with LukS-PV for 24 h and then incubated with 10 μM MG132 for 5 h before harvesting. The cells were lysed in IP lysis buffer (catalog no.: P0013; Beyotime) containing a protease inhibitor cocktail. Cell lysates were immunoprecipitated with anti-β-catenin antibody overnight at 4 °C and incubated with 20 μl Protein A/G PLUS-Agarose beads for 4 h. The beads were washed, and the enriched protein samples were separated by SDS-PAGE and subjected to WB with anti-β-catenin and antiubiquitin antibodies. The related antibodies and reagents used are listed in Table S2.

### Site-directed mutation of the β-catenin locus

Total RNA was extracted from HepG2 cells, and wildtype β-catenin mRNA was amplified by RT–PCR. The recovered DNA was digested with the appropriate enzymes and cloned into the eukaryotic expression vector pcDNA3-1(−). With this expression plasmid as the template, the lysine 49 (K49) residue of the β-catenin was mutated to glutamine to produce an acetylation-mimicking mutant (K49Q) using the QuickMutation Plus Site-Directed Mutagenesis Kit. The K49 residue was also mutated to arginine to generate an acetylation-resistant mutant (K49R) of β-catenin. After confirmational sequencing, these mutant plasmids were used in subsequent experiments.

### Cell proliferation assay

HCC cells (2 × 10^3^ cells/well) were seeded in 96-well plates after treatment with LukS-PV, HDAC7 expression plasmid DNA, or shRNA for 24 h. Cell proliferation was assessed using CCK8 reagent for a 2 h incubation at 37 °C. The absorbance of each well was measured at 450 nm using an ELx808 microplate reader (BioTek).

### Colony formation assay

HCC cells (500 cells/well) were added to 12-well culture plates and incubated at 37 °C for 7 to 10 days. After washing twice with PBS, the cells were fixed with 70% ethanol for 15 min and stained with 0.5% crystal violet (catalog no.: C0121; Beyotime). The numbers of colonies were counted using ImageJ software (was manufactured by National Institutes of Health).

### EdU assay

The proliferation capacity of HCC cells after transfection with shRNA or plasmid DNA, or treatment with LukS-PV, was detected using a kFluor488-EdU cell proliferation assay kit. The EdU was labeled with green fluorescent kFluor488 dye. Cells were seeded at 5000 cells/well in 96-well plates and incubated with LukS-PV or shRNA for 24 h. A 50 nM EdU working solution was then added and incubated for an additional 2.5 h. Hoechst 33342 was used to counterstain nuclei for 30 min in the dark. Finally, EdU-positive cells were observed and counted under a fluorescence microscope.

### Cell scratch assay

Cell scratch assay was conducted following a previously described protocol (25). The cells were inoculated into six-well plates at a density of 90% and cultured overnight in an incubator. A scratch perpendicular to the cell layer was created using a 10 μl pipette tip. Subsequently, the cells were rinsed twice with PBS to remove loose cells, and fresh serum-free medium was added to continue the culture. Images were captured at 0 h and 48 h after the scratch assay with an inverted microscope to assess migration distance at the scratch region. The percentage of relative migration = (gap width at 0 h gap width at 48 h)/(gap width at 0 h) × 100%. Three duplicated wells were set in each well, and the experiment was repeated three times.

### Quantitative proteomics sequencing

Quantitative proteomics sequencing was conducted following a previously described protocol (24). In brief, cells were treated with LukS-PV for 24 h and then sonicated three times in lysis buffer. After collecting the supernatant, the proteins were reduced with 5 mM dithiothreitol and alkylated with 11 mM iodoacetamide and the urea in the lysis buffer. Finally, trypsin was added for overnight protein digestion after which the peptides were reconstituted in 0.5 M triethylamine bicarbonate and labeled using the TMT kit/iTRAQ kit. The peptides were isolated and purified by high pH, reverse-phase HPLC using an Agilent 300 Extend C18 column. The peptides were added to a nanoelectrospray ionization source followed by tandem mass spectrometry (MS/MS) using a Q Exactive Plus mass spectrometer coupled to an ultraperformance LC system. The resulting LC–MS/MS data were processed using the MaxQuant proteomics software package and search engine (V.1.5.2.8).

### Immunopurification and MS

The HepG2 cells, which exhibit stable expression of β-catenin, were lysed in radioimmunoprecipitation assay lysis buffer and centrifuged at 10,000*g* for 10 min at 4 °C. IgG and the anti-β-catenin primary antibody were added to the lysates. After overnight incubation at 4 °C, 30 μl protein A/G agarose beads were added to the lysates and incubated at 4 °C for 3 h. The beads were washed three times with lysis buffer, followed by boiling at 100 °C for 10 min in 2× SDS loading buffer. Proteins in the supernatant were analyzed by WB. These proteins were also separated by SDS-PAGE and stained with Coomassie brilliant blue. After staining the gel, the entire lane was removed and sent to General Biotechnology for MS analysis. IP and LC–MS/MS analyses results are listed in Table S1.

### Immunofluorescence staining

The HepG2 cells were seeded on 12-mm diameter round glass cover slips in 6-well plates. After overnight incubation at 37 °C, the cells were washed three times with PBS, and fixed with 4% paraformaldehyde for 15 min. The fixed cells were washed with PBS and permeabilized with 0.5% Triton X-100 for 20 min. After incubation for 60 min in blocking buffer, the cells were incubated with anti-β-catenin antibody overnight at 4 °C. After three washes with PBS, the cells were incubated with the secondary antibody, FITC-conjugated goat anti-rabbit IgG for 60 min at room temperature. The cells were washed and counterstained with 4′,6-diamidino-2-phenylindole to visualize the nuclei. Cells on cover slips were mounted onto glass slides, and images were visualized with a Zeiss LSM800 confocal microscope.

### Xenograft tumor models in nude mice

BALB/c nude mice (4 weeks old) were purchased from Anhui Provincial Animal Center. For xenografts, 200 μl Hun7 cell suspension with approximately 5 × 10^6^ cells was subcutaneously injected. Treatments were performed by intraperitoneal injection of LukS-PV or PBS 72 h later for 10 days. Tumor size was recorded every 4 days. Then 26 days after tumor inoculation, all experimental mice were sacrificed. Animal experiments were authorized by the Ethics Committee of the First Afﬁliated Hospital of USTC.

### Immunohistochemical staining

Tumor tissues from mice were fixed in 4% formaldehyde solution, embedded in paraffin, and sliced into continuous sections of 4-μm thickness. Following deparaffinization of the tissue sections, endogenous peroxidase was blocked with 3% peroxide, and antigens were retrieved by microwave heating. Sections were blocked in a buffer containing 5% bovine serum albumin and 0.1% Triton X-100 and incubated with primary antibodies overnight at 4 °C. After washing three times with PBS, the tissue slides were treated with a nonbiotin horseradish peroxidase detection system, following the manufacturer’s instructions.

### Statistical analysis

Results are expressed as the means ± SD of at least three independent experiments. Differences between multiple groups were checked using one-way ANOVA. Differences between two groups were analyzed by a two-tailed unpaired Student’s *t* test. A value of *p* < 0.05 was considered statistically significant, and *p* < 0.01, *p* < 0.001, and *p* < 0.0001 indicated strongly significant differences. Pearson’s correlation analysis was performed to determine the correlation between two variables. The graphs were generated using GraphPad Prism 8 (GraphPad, Inc).

## Data availability

Derived raw data supporting the results of this study are available from the corresponding author on request.

## Supporting information

This article contains supporting information.

## Conflict of interest

The authors declare that they have no conflicts of interest with the contents of this article.

## Supporting information

Supporting Table S1

Revised Supporting Information
