# Supplementary material for: Toxin protein LukS-PV targeting complement receptor C5aR1 inhibits cell proliferation in hepatocellular carcinoma via the HDAC7–Wnt/β-catenin axis
Source: J Biol Chem. 2024 Dec 28;301(2):108148. doi: 10.1016/j.jbc.2024.108148 (PMC11910327; doi:10.1016/j.jbc.2024.108148)
Supplement: Revised Supporting Information [file mmc2.docx]

**Supporting Information**

**Toxin protein LukS-PV** **targeting complement receptor C5aR1 inhibits** **cell proliferation in hepatocellular carcinoma via** **the** **HDAC7-****Wnt/****β-catenin** **axis**

Running Title: LukS-PV anti-HCC via HDAC7-Wnt/β-catenin axis by targeting C5aR1

Lan Shi^1^, Shanshan Zhang^2^, Gan Liu^1^, Zhengchao Nie^1^, Pengsheng Ding^1^, Wenjiao Chang^1^, Yuanyuan Dai^1^, Xiaoling Ma^1,3*^

**Figure S1. C5a targeting** **C5aR1 promotes the proliferation of HCC cells.**

(A) CCK-8 assay was performed to detect cell viability of HepG2 and Bel-7402 cells treated with different concentrations of C5a for 1, 2, 3, and 4 day.

(B) Colony formation assay was conducted to determine the clonogenic ability of HepG2 and Bel-7402 cells treated with different concentrations of C5a for 14 days.

(C) CCK-8 assay was performed to detect cell viability of Bel-7402 cells treated with different concentrations of LukS-PV for 1, 2, 3, and 4 day.

(D) Colony formation assay was conducted to determine the clonogenic ability of Bel-7402 cells treated with different concentrations of LukS-PV for 14 days.

**Figure S2. LukS-PV targeting C5aR1 inhibits the proliferation of HCC cells by downregulating Wnt/**β**-catenin signaling.**

(A) RNA sequencing was performed in HepG2 cells treated with LukS-PV or PBS.

(B) Volcano plot of different expression genes upon LukS-PV or PBS treatment in HepG2 cells.

(C) Quantitative proteomics sequencing of HepG2 cells treated with LukS-PV or PBS showing changes in CTNNB1 expression levels.

(D) Western blotting analysis the levels of β-catenin, C-myc and cyclinD1 expression in Bel-7402 cells treated with different concentrations of LukS-PV for 24h. GAPDH was used as the control.

(E) Hep3B cells were treated with LukS-PV, or combination of LukS-PV and OE-C5aR1. The protein expression levels of C5aR1, β-catenin, C-myc and cyclinD1 were detected by western blotting.

(F) HepG2 cells pretreated with Wnt3a stimulation and then treated with LukS-PV, the protein expression levels of β-catenin, C-myc and cyclinD1 were detected by western blotting. Relative band intensities (compared to GAPDH) were measured using Image J.

**Figure S3. LukS-PV inhibits HCC cell proliferation by upregulating the acetylation of β-catenin to promote its degradation.**

1. Western blotting analysis the levels of acetylated and phosphorylated β-catenin, C-myc, cyclinD1, and β-catenin in HepG2 cells transfected with the β-catenin K49Q and K49R mutant expression plasmids.

**Figure S4.** **HDAC7 mediates the deacetylation of β-catenin to promotes HCC cell migration.**

(A) Western blotting analysis the levels of β-catenin, C-myc and cyclinD1 in HepG2 cells treated with different concentrations of SAHA for 24h.

(B-C) The mobility of HepG2 cells were examined by scratch assays after transfected OE-HDAC7, and the percentage of relative migration was calculated.

(D-E) The mobility of Bel-7402 cells were examined by scratch assays after knocking down HDAC7 with shRNA, and the percentage of relative migration was calculated.

(F-G) The mobility of HepG2 cells were examined by scratch assays after transfected OE-HDAC7 combine with the β-catenin K49Q and K49R mutant, and the percentage of relative migration was calculated.

**Figure S5. LukS-PV downregulates the high expression of HDAC7 by targeting C5aR1 in HCC cells.**

(A) Hep3B cells were treated with LukS-PV, or combination of LukS-PV and OE-C5aR1. The protein expression levels of C5aR1 and HDAC7 were detected by western blotting.

(B) Western blotting analysis of HDAC7 protein expression levels in HCC cells treated with different concentrations of C5a.

**Supplementary Table 2. Key Materials in this study**

| **Category** | **Source** | **Cat. No.** |
| --- | --- | --- |
| **Antibodies** | | |
| β-catenin | Proteintech | 66379-1-Ig |
| CyclinD1 | Proteintech | 60186-Ig |
| C-myc | Proteintech | 67447-1-Ig |
| C5aR1 | Proteintech | 21316-1-AP |
| HDAC7 | Cell signaling technology | 10831 |
| acetylated-lysine | Cell signaling technology | 9441 |
| phospho-β-catenin | Cell signaling technology | 9564 |
| acetyl-β-catenin | Cell signaling technology | 9030 |
| GAPDH | Abclonal | AC002 |
| Goat Anti-Rabbit IgG-HRP | Abclonal | AS014 |
| Goat Anti-Mouse IgG-HRP | Abmart | AS003 |
| Goat Anti-Rabbit IgG-FITC | ZSGB-BIO | ZB-2301 |
| **Chemicals** | | |
| Cycloheximide | Beyotime | SC0353 |
| MG132 | Beyotime | S1748 |
| Protein A/G PLUS-Agarose | Santa Cruz | sc-2003 |
| Lipofectamine 2000 | Invitrogen | 11668019 |
| SYBR Green Master Mix | TaKaRa | RR820A |
| RIPA buffer | Beyotime | P0013B |
| 4% paraformaldehyde | BIOMIKY | BL014A |
| Triton X-100 | Sigma | T9284 |
| DAPI | Beyotime | C1005 |
| peroxide | Sigma | 88597 |
| bovine serum albumin BSA | BIOMIKY | BR008B |
| **Critical Commercial Assays** | | |
| RNeasy Mini Kit | QIAGEN | 74106 |
| TruSeq RNA Sample Preparation Kit | Illumina | RS-122-2001 |
| PrimeScript RT reagent kit | TaKaRa | RR036A |
| BCA Protein Assay Kit | Beyotime | P0012 |
| TRIzol Reagent | Invitrogen | 15596026 |
| QuickMutation Plus Site-Directed Mutagenesis Kit | Beyotime | D0208S |
| Cell Counting Kit-8 (CCK8) reagent | BIOMIKY | BL001B |
| kFluor488-EdU cell proliferation assay kit | KeyGEN | KGA9602 |

**Supplementary Table 3. Primers used in this study.**

| **Primers sequence（Primers for qRT-PCR）** | |
| --- | --- |
| β-catenin (*CTNNB1*)*-F* | AAGTTCTTGGCTATTACGACA |
| β-catenin (*CTNNB1*)*-R* | ACAGCACCTTCAGCACTCT |
| *C-myc-F* | GGAACTATGACCTCGACTACGAC |
| *C-myc-R* | ACCATGTCTCCTACAGTAGCTC |
| *HDAC7-F* | AACGGCACGT-GCTCCTCTA |
| *HDAC7-R* | TCCTGTAAGCTTCCCGTTATCC |
| *GAPDH-F* | GGAGCGAGATCCCTCCAAAAT |
| *GAPDH-R* | GGCTGTTGTCATACTTCTCATGG |
| **Primers sequence（Primers for PCR）** | |
| HDAC7-sh1 | CCA CUUUGCCCAGUCCUUATT |
| HDAC7-sh2 | GCUUCAUUCCUCCAAUGCATT |
| HDAC7-sh3 | GCUAAA GAAUGGUUUCGCUTT |
| C5aR1-sh1 | UGGUGGGAGUGCUGGGCAATT |
| C5aR1-sh2 | CUGAAGAGUCCGUGGUUAGTT |
| C5aR1-sh3 | UCAAGGUGGUGGUGGCAGUTT |
